# Case Report: Full-Thickness Forehead Burn Over Indwelling Titanium Hardware Resulting From an Aberrant Intraoperative Electrocautery Circuit

**Published:** 2007-11-20

**Authors:** Gerhard S. Mundinger, Shai M. Rozen, Benjamin Carson, Robert S. Greenberg, Richard J. Redett

**Affiliations:** Johns Hopkins School of Medicine, Baltimore, MD; Division of Plastic and Reconstructive Surgery, Johns Hopkins School of Medicine, Baltimore, MD; Department of Neurosurgery, Johns Hopkins School of Medicine, Baltimore, MD; Department of Anesthesia and Critical Care Medicine, Johns Hopkins School of Medicine, Baltimore, MD; Division of Plastic and Reconstructive Surgery, Johns Hopkins School of Medicine, Baltimore, MD

## Abstract

**Objective:** This study aims to contextualize an unintended intraoperative electrocautery burn that occurred on our service within the spectrum of all intraoperative electrocautery burns. **Methods:** A case report of the incident was drafted, and the relevant literature present in PubMed and industry publications was reviewed. **Results:** Intraoperative electrocautery burns can be divided into 4 categories: (1) direct contact burns resulting from inappropriate operator use of the active electrode, (2) burns at the grounding electrode site due to improper attachment or placement, (3) burns resulting from electrode heating of pooled solutions, and (4) burns occurring outside the operative field as a result of circuits generated between the active electrode and an alternate grounding source. We herein report an unintended intraoperative electrocautery burn of the fourth category. An aberrant intraoperative circuit utilized previously placed in-dwelling titanium plating in the patient's right brow as the grounding electrode, resulting in 3 × 3-cm full-thickness skin necrosis overlying the site of hardware implantation. **Conclusions:** Literature recommendations to reduce this type of electrocautery burn suggest avoiding grounding pad placement on the forearm and lateral thigh, although further investigation is needed to determine optimal grounding electrode placement with respect to known indwelling hardware.

## CASE REPORT

**Figure 1:**
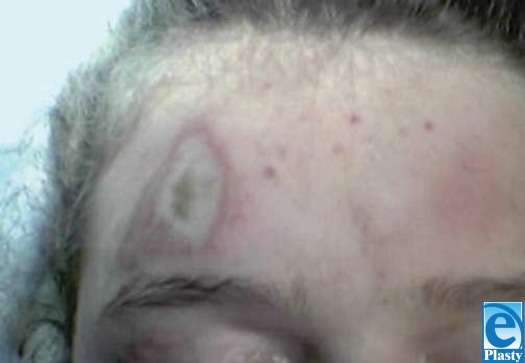
Photograph of the burn site on the day of surgery

**Figure 2:**
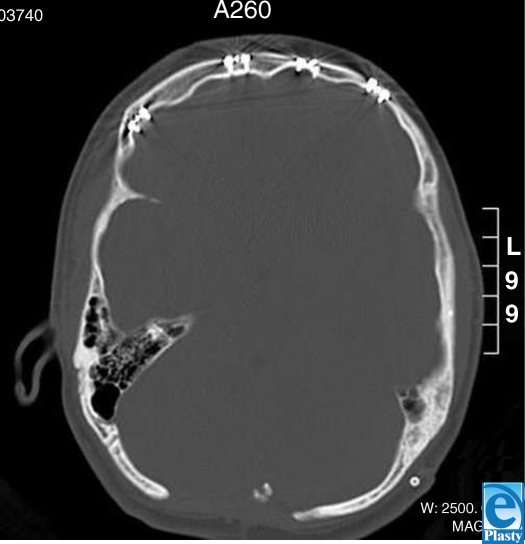
Computed tomography scan and plain film of the patient demonstrating the location of indwelling titanium hardware placed at prior surgery. The burn occurred on the right forehead directly over the most lateral piece of hardware visualized in the coronal section

**Figure 3:**
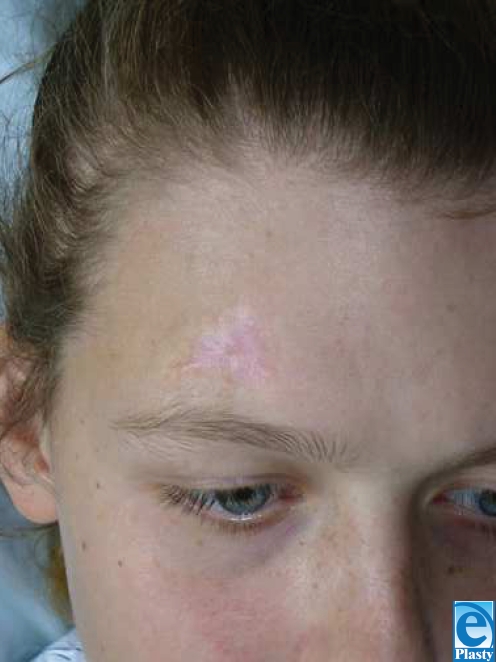
Photograph of the burn site at 3-month follow-up demonstrating complete injury resolution

The patient is 14-year-old girl with a complex medical and surgical history notable for Chiari malformation, sagittal sinus thrombosis, hydrocephalus, secondary craniosynostosis, and seizure disorder. Of note, several titanium plates remained in her left and right frontal, temporal, and parietal regions following full cranial expansion in June 1993 with subsequent plate and screw revision in June 1994. She had multiple ventriculoperitoneal and lumbarperitoneal shunt revisions beginning in 1995, and underwent cervicomeduallry decompressions in August 1998 and January 2004. In March 2005, she presented with recurrent symptoms of severe headache and vomiting. Magnetic resonance imaging revealed a collection of scar tissue at the cervicomedullary junction. Accordingly, she underwent cervicomedullary exploration and decompression for her Chiari malformation from an occipital approach. She was placed in the prone position with her head resting on a well-padded Mayfield horseshoe. One grounding pad was applied on the left thigh. Her surgery proceeded without incident. Total operating time was 1.5 hours. When she was turned over at the conclusion of surgery, one area of erythema was noted on the right side of her forehead. Plastic surgery was considered. A swollen 3 × 3-cm triangular area of brown dermis with visibly coagulated veins was noted above her right brow (Fig 1). Two zones, the inner white and outer red, extended concentrically from this area of coagulation and corresponded to the exact location of her frontal hardware as seen on computed tomography scan (Fig 2). The burn was treated with Hydrogel and Duoderm. Follow-up at 3 months revealed complete healing of the burn site (Fig 3).

## DISCUSSION

While incorporating the patient's body into a high-frequency electrical circuit, modern electrosurgical devices are able to selectively cauterize and volatilize tissues by dissipating current density as it leaves the patient's body, thereby avoiding unintended thermal injury. This is accomplished by a large grounding, or “indifferent” electrode that completes the circuit between patient and electrocautery device.^1^ Since the introduction of the first successful unipolar electrocautery by William T. Bovie in 1928, and the bipolar electrocautery by Greenwood in 1939, a number of thermal complications have been realized and reported in the literature.^2^,^3^ Although the overall incidence of recognized aberrant electrocautery burns is between 1 and 2 patients per thousand operations, reports of unintended electrosurgical thermal injury are rare.^4^ They can be divided into 4 categories:

1. Direct contact burns in the operative field result from imprecise active electrode use. An unintentional oral commisure burn resulting form improper bipolar electrocautery technique during tonsillectomy was described in the otolaryngology literature, and attributed to operator ignorance and inexperience.^3^ More common than other types of electrocautery injury, operative field burns are underreported.
2. Improper placement or attachment of the grounding electrode can lead to burns at the site of indifferent electrode attachment. While modern electrodes have been designed to minimize this complication, burns at the site of indifferent electrode attachment still occur and can be severe.^5^ Recent reports of this complication have been published in the interventional cardiology and surgery literature.^4^,^6^,^7^ Between December of 1996 and April of 1998, the Food and Drug Administration received 628 reports of grounding pad burns.^8^ A noncontact electrosurgical grounding device has recently been developed for use in severe burn cases where available sites for grounding electrode placement are sparse.^9^ Widespread adoption of this device could potentially eliminate this complication.
3. Electrosurgical units can heat pooled solutions, resulting in thermal injury. Burns have been attributed to solution heating by both the active and the indifferent electrode.^4^,^10^,^11^ A case recently reported in the cardiac surgery literature describes partial and total thickness burns to 22% of total body surface area, ultimately contributing to patient mortality.^11^ Local burns attributed to electrocautery heating of surgically injected solution have been reported in the orthopedic arthroscopic literature.^12^,^13^
4. Aberrant intraoperative circuits can be generated by monitoring or operative equipment contacting the patient's body, leading to thermal injury at sites of contact remote from the operative field. Such “alternate-site” or “capacitative coupling” burns have long been recognized, and occur when current preferentially passes from the active electrode through a grounding site other than the indifferent electrode. Burns resulting from aberrant circuits have been reported at sites of electrocardiographic lead placement, temperature probe insertion, uninsulated surgical table contact with the patient, intra-arterial line placement, motor-evoked potential monitoring electrode placement, and electroencephalogram electrode placement.^4^,^14^–^20^ In addition, when monopolar and bipolar cautery are used concurrently, current generated from monopolar cautery activation can ground through bipolar tynes left in direct contact with the patient, leading to documented intraoperative burns.^21^ Alternate-site burns arise from highly variable accidental circumstances surrounding grounding electrode placement and function, and are influenced by electric fields generated by other operating theatre devices.^4^,^22^ In addition to alternate site burns, pressure sores and chemical burns should be considered in the differential diagnosis of newly discovered intraoperative or perioperative lesions.^23^

Our case is an example of the fourth type of intraoperative electrosurgical thermal injury. Despite an appropriately attached grounding pad, right frontal indwelling titanium hardware from previous craniosynostosis revision served as an alternate indifferent electrode, generating a focused aberrant circuit passing through this site. This circuit heated the hardware, resulting in thermal injury to surrounding tissues. The burn was typical in appearance for alternate site burns, with a central area of full-thickness necrosis surrounded by concentric zones of pallor and, more peripherally, hyperemia.^23^ While both monopolar and bipolar electrocautery devices were used intraoperatively, current flowing between bipolar cautery tynes minimally disperses through the body, making capacitative coupling burns from bipolar cautery activation exceedingly unlikely.^4^ At no time were the bipolar tynes in contact with the patient when the monopolar cautery was activated. Thus, current generated by monopolar cautery caused this intraoperative burn. To our knowledge, this is the first report of remote thermal injury caused by an aberrant intraoperative current grounding through indwelling metallic hardware.

Based on this case report and review of the relevant literature, a number of suggestions to reduce alternate site burns can be made. Recommendations to reduce unintended intraoperative electrocautery burns have been previously documented in the literature and include the following: using isolated electrosurgical units, avoiding or minimizing contact between the patient and operative instruments and theatre devices, avoiding activation of the electrocautery when not in contact with tissue, proper indifferent electrode attachment, and optimal placement of the indifferent electrode.^3^,^4^,^11^,^19^,^20^,^24^

There is little available data addressing the relationship between the location of grounding pad placement relative to surgical site and the effect this placement has on intraoperative dispersive electrode function. One study attempted to address this issue by measuring body deep tissue impedance between various surgical sites and various grounding pad placement sites.^22^ This study found that, in head and neck procedures, placing the indifferent electrode on the mid-sternum, thoracic spine at T6, lateral chest wall mid-way between the axilla and 12th rib, or lower anterior abdominal quadrant, resulted in the lowest, and roughly equivalent, body deep tissue impedance values. These results imply that, in craniofacial procedures, preferentially placing grounding pads at these sites in the order listed may reduce the risk of alternate-site burns. In addition, the study indicates that indifferent electrode placement on the thigh and forearm should be avoided, as these sites generated maximal impedance levels. Considering the results of this study, the indifferent electrode in our case was malpositioned in its placement on the left thigh, and may have contributed to thermal injury overlying the titanium implant.

Recommendations with respect to grounding pad placement relative to metal prostheses are anecdotal. Electrosurgical unit manufacturers recommend avoiding grounding pad placement immediately adjacent to or overlying indwelling hardware, as the high resistance of scar tissue incasing prostheses can generate increased temperatures, possibly resulting in thermal injury to surrounding tissues.^25^ This recommendation has been adopted in the perioperative nursing literature.^24^ However, this consideration must be weighed against evidence that impedance, and thus the likelihood of alternate-site burns, seems to be reduced by grounding pad placement closer to the operative field.^22^ As we have reported a case in which an alternate-site burn occurred overlying indwelling titanium plating, such discussion is no longer an esoteric undertaking, and more investigation is needed to definitively address the issue of grounding pad placement relative to implanted metal prostheses.

## SUMMARY

Intraoperative electrocautery burns, although relatively common, are infrequently reported in the literature. They can be divided into 4 categories: (1) direct contact burns in the operative field resulting from inappropriate operator use of the active electrode, (2) burns at the grounding electrode site due to improper indifferent electrode attachment or placement, (3) burns resulting from either the active or indifferent electrode heating pooled solutions, and (4) burns occurring outside the operative field as a result of aberrant intraoperative circuits generated between the active electrode and an alternate grounding source (ie, “alternate-site,” or “capacitative coupling” burns). We herein report an unintended intraoperative electrocautery burn of the fourth category, in which an aberrant circuit utilized previously placed indwelling titanium plating in the patient's right brow as the grounding electrode. This circuit resulted in 3 × 3-cm full-thickness skin necrosis overlying the site of hardware implantation. To our knowledge, this is the first report of its kind in the English language literature. Such alternate-site burns result from highly variable and currently unpredictable intraoperative circumstances. Literature recommendations to reduce intraoperative electrocautery burns suggest avoiding grounding pad placement on the forearm and lateral thigh. Considering alternate-site burns, further investigation is needed to determine optimal grounding electrode placement with respect to both operative site and known indwelling hardware.
